# Eltrombopag treatment of a dog with idiopathic aplastic pancytopenia

**DOI:** 10.1111/jvim.15738

**Published:** 2020-02-25

**Authors:** Darren Kelly, Valerie Lamb, Florence Juvet

**Affiliations:** ^1^ Southern Counties Veterinary Specialists Ringwood UK

**Keywords:** anemia, bone marrow, neutropenia, thrombocytopenia, thrombopoietin

## Abstract

Idiopathic aplastic pancytopenia is an uncommon disease in dogs which results in pancytopenia and for which an immune‐mediated etiology is suspected. A small number of affected dogs reported in the veterinary literature have responded to immunosuppressive medication but the prognosis generally is considered poor with a reported mortality rate of 80%. Reported response rates to immunosuppression alone in affected people are low with overall and complete responses of 65 and 10%, respectively. With the addition of eltrombopag, an orally available thrombopoietin receptor agonist, reported overall and complete response rates in people increase to 94 and 58%, respectively. Herein, we report the use of eltrombopag in a dog with idiopathic aplastic pancytopenia. Eltrombopag was started after no response was seen to treatment with prednisolone and cyclosporine. Complete remission was achieved after the addition of eltrombopag and was sustained after stopping the medication.

## 1 INTRODUCTION

Idiopathic aplastic pancytopenia is characterized by cytopenias in multiple cell lines with hypoplasia of the bone marrow and replacement with adipose tissue with or without fibrosis and for which an inciting cause cannot be identified.^1^ Aplastic pancytopenia can be caused by infectious agents, drugs, and toxins but a diagnosis of idiopathic aplastic pancytopenia accounts for <1% of bone marrow samples evaluated in dogs.^1^ In the absence of a known inciting agent, the term idiopathic is used but increasing evidence suggests an immune‐mediated pathogenesis.^1^ In the veterinary literature, only a few cases of idiopathic aplastic pancytopenia have been reported, with mortality rates of 66%‐80% and with most animals dying or being euthanized within 2‐3 weeks of diagnosis.^1^, ^2^, ^3^ Given the suspicion of an immune‐mediated etiology in dogs, trial treatment with immunosuppressive drugs has been recommended and a small number of reported cases have responded to immunosuppressive treatment.^1^, ^2^, ^3^, ^4^, ^5^ In human patients, immunosuppression with cyclosporine is considered the cornerstone of treatment for aplastic pancytopenia, but overall and complete response rates are low at 65 and 10%, respectively.^6^ In human patients with aplastic pancytopenia, the addition of the thrombopoietin receptor agonist eltrombopag to immunosuppressive treatment leads to improved outcomes with overall and complete response rates of 94 and 58%, respectively.^7^

## 2 CASE DESCRIPTION

A 1‐year‐old male neutered Border Collie was referred for investigation and treatment of pancytopenia. The dog was presented to the primary care veterinarian with a 2‐day history of lethargy and anorexia. There was no known access to toxins and the dog had never traveled outside of the United Kingdom. The dog was found to be pyrexic and a CBC showed pancytopenia. At the time of presentation to the referral hospital, the dog was quiet and pyrexic (39.5°C). Petechial hemorrhages were visible on the ventral abdominal skin and the oral mucosa. Mucous membranes were slightly pale.

The initial CBC showed pancytopenia. Moderate, normocytic (mean cell volume, 66.9 fl; reference range, 60‐80 fl), normochromic (mean cell hemoglobin concentration, 35 g/dL; reference range, 30.8‐37.0 g/dL), non‐regenerative anemia (red blood cell count, 3.38 × 10^12^/L; reference range, 5.0‐8.5 × 10^12^/L and reticulocyte count 3.35 × 10^9^/L), severe thrombocytopenia (platelet count, 2 × 10^9^/L; reference range, 160‐500 × 10^9^/L), and severe neutropenia (neutrophil count, 0.08 × 10^9^/L; reference range, 3.0‐11.5 × 10^9^/L) were present. These findings were confirmed on blood film examination. A Coombs' test (direct antiglobulin test) was weakly positive at 1/16 (<1:64 not considered clinically relevant). Serum biochemistry showed mild hypoalbuminemia (22 g/L; reference range, 26‐38 g/L) and mild increases in serum alanine aminotransferase (88 U/L; reference range, 0‐25 U/L) and alkaline phosphatase (211 U/L, reference range, 0‐50 U/L) activity. The hypoalbuminemia was thought to indicate a negative acute phase response. The mild increases in serum hepatobiliary enzyme activity were considered most likely to represent a reactive hepatopathy, and because the changes were mild and the liver appeared normal on abdominal ultrasound examination (see below), further investigation of these biochemical changes did not seem warranted. No abnormalities were identified on urinalysis. Serology for Ehrlichia (SNAP 4Dx, IDEXX Laboratories Inc, Maine) and Leishmania (Quantitative ELISA, VPG SYNLAB, United Kingdom) spp was negative. No abnormalities were seen on thoracic radiographs or abdominal ultrasound examination. A bone marrow aspirate and core biopsy were performed. Aspiration cytology confirmed poorly cellularity with only small to moderately sized aggregates of adipocytes and small numbers of spindle cells. Among the aggregates, occasional plasma cells, hemosiderophages, and lymphocytes were observed. Histologic evaluation of the core biopsy sample showed well organized, mature bone trabeculae. Within the marrow spaces, adipose tissue and streams of fibroblasts were present, supported by collagenous stroma. Aggregates of erythrocytes were admixed with occasional hemosiderophages. Scattered small lymphocytes and plasma cells were observed. The histological diagnosis was marked, diffuse bone marrow hypoplasia with mild fibrosis and mild inflammation. An immune‐mediated etiology was suspected based on the results of the diagnostic investigation.

The dog was hospitalized for 11 days and treated with IV fluid therapy and amoxicillin‐clavulanate (Augmentin, GlaxoSmithKlein, United Kingdom) 20 mg/kg IV q8h. Based on the assumption of an immune‐mediated etiology, the dog was treated with dexamethasone (Colvasone, Norbrook, United Kingdom) 0.4 mg/kg IV q24h and cyclosporine (Atopica, Novartis, Switzerland) 5 mg/kg PO q12h. The dog required 2 transfusions of type‐appropriate and cross‐matched packed red cells on days 2 and 10 of hospitalization because the packed cell volume decreased to 15 and 16%, respectively, and the dog developed generalized weakness and tachycardia. By day 10 of hospitalization, no improvement had occurred with regard to platelet, neutrophil, or reticulocyte counts. Eleven days after first presentation, the dog was started on eltrombopag (Revolade, Novartis, Switzerland) 1.25 mg/kg PO q24h and was discharged on prednisolone (Prednicare, AnimalCare, United Kingdom) 2 mg/kg PO q24h, cyclosporine 5 mg/kg PO q12h, and amoxicillin‐clavulanate 20 mg/kg PO q12h.

An initial response to the treatment was seen 19 days after starting eltrombopag (30 days after initial presentation) and the CBC showed red blood cell count 4.00 × 10^12^/L (reference range, 5.0‐8.5 × 10^12^/L), reticulocyte count 88 × 10^9^/L, mean cell volume 68.8 fl (reference range, 60‐80 fl), mean cell hemoglobin concentration 34.2 g/dL (reference range, 30.8‐37.0 g/dL), platelet count 52 × 10^9^/L (reference range, 160‐500 × 10^9^/L), and neutrophil count 1.88 × 10^9^/L (reference range, 3.0‐11.5 × 10^9^/L). At this point, the antibiotics were discontinued. Complete hematological response, with normalization of circulating neutrophil and platelet counts and appearance of reticulocytosis, was seen 33 days after starting treatment with eltrombopag (44 days after initial presentation). The CBC at this point showed red blood cell count 4.39 × 10^12^/L (reference range, 5.0‐8.5 × 10^12^/L), reticulocyte count 180 × 10^9^/L, mean cell volume 72.4 fl (reference range, 60‐80 fl), mean cell hemoglobin concentration 33.0 g/dL (reference range, 30.8‐37.0 g/dL), platelet count 161 × 10^9^/L (reference range, 160‐500 × 10^9^/L), and neutrophil count 6.38 × 10^9^/L (reference range, 3.0‐11.5 × 10^9^/L). The dosage of prednisolone was tapered and subsequently discontinued after approximately 3 months. The dosage and frequency of cyclosporine was unchanged for the first 5 months and the frequency of administration then was decreased to once daily for a further 1 month and then discontinued. The eltrombopag was continued for a total of 2 months and then discontinued. The dog remained clinically well and in remission after 10 months of follow‐up. Aside from adverse effects attributable to corticosteroid administration (eg, polyuria, polydipsia, and polyphagia), all of which resolved after discontinuing the corticosteroids, no other adverse effects were reported.

## 3 DISCUSSION

Eltrombopag is an orally available thrombopoietin receptor agonist which was first approved for use in human patients with refractory idiopathic thrombocytopenia purpura.^8^ After initiation of its use in the clinical setting, eltrombopag was found to have bi‐lineage and tri‐lineage bone marrow stimulatory effects and, in recent years, several studies have shown clinically relevant responses to eltrombopag in humans with aplastic pancytopenia.^6^, ^7^, ^9^, ^10^ The mechanism by which this thrombopoietin receptor agonist stimulates multi‐lineage recovery is not completely understood but is thought to be related to stimulation and proliferation of primitive hematopoietic stem cells and other progenitor cells that may express the thrombopoietin receptor.^11^ Aside from patients with refractory disease, eltrombopag has been used alongside immunosuppression in treatment‐naive human patients with severe aplastic pancytopenia and has been shown to accelerate cell count recovery. One study described the use of eltrombopag for a set period of 10 weeks in treatment‐naive human patients and found a response rate of 87% at 3 months after starting treatment and response rates were sustained at 6 months, 3 months after discontinuing eltrombopag.^6^ The duration of eltrombopag treatment used in our case (2 months) was based on this study and, as also was found in this study of human patients, clinical remission in the dog reported here was sustained after discontinuing the eltrombopag. The times to initial and complete hematological response in this dog were 30 and 44 days, respectively. This response is similar to a study in humans investigating the combined use of eltrombopag and cyclosporine in treatment‐naive patients with pancytopenia, which found a median response time of 35 days.^6^ Multiple studies have shown sustained responses when eltrombopag treatment is discontinued, which has led to the suggestion that starting eltrombopag early in treatment‐naive patients may be warranted because of its proposed ability to salvage and cause expansion of residual hematopoietic stem cells.^6^, ^10^

Ours is the first report of the use of eltrombopag in a dog. Idiopathic aplastic pancytopenia in dogs generally carries a poor prognosis with a reported 21‐day mortality rate of 80%.^1^ The high mortality rate reported in dogs likely reflects poor response to immunosuppressive treatment, similar to the poor response seen when immunosuppressive treatment is used alone in human patients. The decision to start treatment with eltrombopag in this case was made when no response to immunosuppressive treatment was seen and given the relatively high response rates to eltrombopag reported in humans. In this case, the dog responded to treatment with immunosuppressive medication and eltrombopag with sustained remission after discontinuing the eltrombopag with 10 months follow‐up. Aside from polyuria, polydipsia, and polyphagia, which resolved after discontinuation of the corticosteroids, no adverse effects were observed. The dosage used in this case was chosen based on the smallest available tablet size and the starting dosage used in children. The main limitation of this case report is that we cannot prove that the eltrombopag was responsible for the response seen, although we feel that this is likely the case. It is possible that the response seen was a response to the immunosuppressive medications in combination with supporting the dog for a sufficiently long period of time. We however feel that this is unlikely given that no response was seen initially when immunosuppressive medications were administered alone and because the time until response seen in this case after starting treatment with eltrombopag closely resembles the median response time reported in human patients. Eltrombopag may be useful for treating aplastic pancytopenia in dogs, but further studies are required to confirm or refute this hypothesis. Although the drug currently is available in many countries worldwide, its cost may make it prohibitive for use in some cases. If used for only a relatively short period of time (eg, 2 months in treatment‐naive patients), the potential for eltrombopag to result in substantially higher sustained response rates when used along with immunosuppressive medications may offset the financial cost of the drug. The purpose of our report is to increase awareness of this drug with the aim of stimulating further investigation into its use in dogs with aplastic pancytopenia, a disease that when treated with the previously described medications (eg, immunosuppressive agents, granulocyte colony stimulating factor, erythropoietin),^12^ typically carries a poor prognosis.

## CONFLICT OF INTEREST DECLARATION

Authors declare no conflict of interest.

## OFF‐LABEL ANTIMICROBIAL DECLARATION

Authors declare no off‐label use of antimicrobials.

## INSTITUTIONAL ANIMAL CARE AND USE COMMITTEE (IACUC) OR OTHER APPROVAL DECLARATION

Authors declare no IACUC or other approval was needed.

## HUMAN ETHICS APPROVAL DECLARATION

Authors declare human ethics approval was not needed for this study.
